# Association of Branched-Chain Amino Acids with Carotid Intima-Media Thickness and Coronary Artery Disease Risk Factors

**DOI:** 10.1371/journal.pone.0099598

**Published:** 2014-06-09

**Authors:** Ruiyue Yang, Jun Dong, Haijian Zhao, Hongxia Li, Hanbang Guo, Shu Wang, Chuanbao Zhang, Siming Wang, Mo Wang, Songlin Yu, Wenxiang Chen

**Affiliations:** 1 The Key Laboratory of Geriatrics, Beijing Hospital & Beijing Institute of Geriatrics, Ministry of Health, Beijing, China; 2 Beijing Hospital and National Center for Clinical Laboratories, Ministry of Health, Beijing, China; Northeast Ohio Medical University, United States of America

## Abstract

**Background:**

Recent studies have determined that branched-chain (BCAAs) and aromatic (AAAs) amino acids are strongly correlated with obesity and atherogenic dyslipidemia and are strong predictors of diabetes. However, it is not clear if these amino acids are capable of identifying subjects with coronary artery disease (CAD), particularly with subclinical atherosclerosis who are at risk of developing CAD.

**Methods:**

Four hundred and seventy two Chinese subjects (272 males and 200 females, 42–97 y of age) undergoing physical exams were recruited at random for participation in the cross-sectional study. Serum BCAAs and AAAs were measured using our previously reported isotope dilution liquid chromatography tandem mass spectrometry method. Bilateral B-mode carotid artery images for carotid intima-media thickness (cIMT) were acquired at end diastole and cIMT values more than 0.9 mm were categorized as increased. Correlations of BCAAs with cIMT and other CAD risk factors were analyzed.

**Results:**

BCAAs and AAAs were significantly and positively associated with risk factors of CAD, e.g., cIMT, BMI, waist circumference, blood pressure, fasting blood glucose, TG, apoB, apoB/apoAI ratio, apoCII, apoCIII and hsCRP, and were significantly and negatively associated with HDL-C and apoAI. Stepwise multiple linear regression analysis revealed that age (β = 0.175, *P*<0.001), log BCAA (β = 0.147, *P*<0.001) and systolic blood pressure (β = 0.141, *P* = 0.012) were positively and independently associated with cIMT. In the logistic regression model, the most and only powerful laboratory factor correlated with increased cIMT was BCAA (the odds ratio of the fourth quartile compared to the first quartile was 2.679; P = 0.009).

**Conclusion:**

BCAAs are independently correlated with increased cIMT. This correlation would open a new field of research in the mechanistic understanding and risk assessment of CAD.

## Introduction

Coronary artery disease (CAD) is the leading cause of death in industrialized countries. Given the availability of effective interventions for delaying or preventing the onset of CAD, early identification of at-risk individuals is particularly crucial [1]. Evaluation with developing tools may improve risk stratification and enhance our understanding of the disease process. Recent advances in liquid chromatography tandem mass spectrometry (LC/MS/MS) allow the acquisition of high-throughput profiles of the metabolic status of whole organisms (eg, metabolomics), which may be particularly useful for the risk assessment of human diseases, such as cardiovascular disease [1], [2].

Branched-chain and aromatic amino acids (BCAAs and AAAs) are noteworthy in the context of experimental and clinical data. It has been suggestedthat these amino acids may both be markers and effectors of insulin resistance [3]–[5] and can predict future diabetes [6], [7]. Both sets of findings have been corroborated by more recent studies using LC-MS-based metabolomics platforms. However, these methods for the analysis of such amino acids in biological samples are time consuming. Our previous study developed a simple, fast and reliable isotope dilution LC/MS/MS method for the measurement of serum BCAAs and AAAs [8]. By using this method, we documented strong associations between certain amino acids and lipid profiles, such as a positive correlation with TG and LDL_b_-C, and a negative correlation with HDL-C and HDL_2_-C [8]. Because the state of dyslipidemia which often precedes type 2 diabetes is associated with an increased risk of developing atherosclerosis and CAD [9], there is great interest in determining whether these amino acids are capable of identifying subjects with coronary artery disease (CAD), particularly with subclinical atherosclerosis who are at risk of developing CAD.

The aims of the current study was to evaluate the associations of BCAAs and AAAs with increased carotid intima-media thickness (cIMT), a well-established marker of subclinical atherosclerosis that can be measured easily and non-invasively [10], and other risk factors of CAD.

## Materials and Methods

### Study samples

This is a cross-sectional study conducted on Chinese subjects. From a group of Beijing residents attending an annual physical examination in a period from August to October, 2011, 472 subjects (272 males and 200 females, 42–97 y of age) were recruited randomly. The sera from fasting blood samples obtained from volunteers were isolated and stored at −80°C until being analyzed.

### Ethics statement

This study has been reviewed and approved by the Ethics Committee of Beijing Hospital, Ministry of Health. All studied individuals had been made aware in writing of the intended use of their blood sample and provided written consent.

### LC/MS/MS measurement for branched-chain and aromatic amino acids

The serum BCAAs [valine (Val), isoleucine (Ile) and leucine (Leu)] and AAAs [tyrosine (Tyr) and phenylalanine (Phe)] were measured using our previously reported isotope dilution LC/MS/MS method [8]. Briefly, 0.05 ml aliquots of calibrators or serum samples were mixed with 0.05 ml of the isotopic labeled internal standard solution and the amino acids were extracted with 0.4 ml of acetonitrile containing 0.1% formic acid, and analyzed using LC/MS/MS. The LC separation was performed on an Agilent 1200 series LC system. Two microliter aliquots of the prepared samples were injected onto a Waters Shield C18 column (3.5 µm, 2.1×150 mm) maintained at 20°C and eluted with a mobile phase of 0.01% formic acid in water-acetonitrile (90∶10) at a flow rate of 0.3 ml/min. An API 4000 triple quadrupole mass spectrometer (Sciex Applied Biosystems) was used for the MS/MS detection. The detection was performed with positive electronic spray ionization (ESI) in multiple reaction monitoring (MRM) mode at a source temperature of 700°C and a voltage of 5500 V. The dwell times were 0.08 s for MRM. Nitrogen was used as the curtain, nebulizer and collision gas at pressures of 50, 60 and 70 psi, respectively. Certain ion transitions for the amino acids and their internal standards were monitored following our previously reported method [8]. The calibration curve was generated using a linear regression of the peak area ratios (y) vs. the amino acid concentrations (x) of the calibrators.

### Carotid intima-media thickness assays

Bilateral B-mode carotid artery images for intima-media thickness were acquired at end diastole (defined as the R wave of an electrocardiogram) using a SDU-1200 ultrasound imager (Shimadzu, Japan) with a linear array 5.5/7.5 MHz probe. The posterior wall of the distal common carotid artery, one centimeter below the bifurcation, was assessed by using the automated IMT analysis system. The maximum of the left and right cIMT readings was used for the analyses. The imaging analysis was performed by an experienced technician blinded to the study participants.

### Other parameters and laboratory assays

A history of diseases and medications and a family history of diseases were recorded using a list of questions. A subject's smoking status was classified as current, former, or never smoker. At the physical examination center, height, weight, waist circumference (WC) and sitting blood pressure were measured. The serum samples were also tested for fasting blood glucose (FBG), total cholesterol (TC), triglycerides (TG), high density lipoprotein cholesterol (HDL-C), low density lipoprotein cholesterol (LDL-C), apolipoprotein (apo) AI, B, CII, CIII and high sensitive C reactive protein (hs-CRP) using assay kits from Sekisui Medical Technologies (Osaka, Japan) on a Hitachi 7180 chemistry analyzer.

### Statistical analyses

Categorical variables are presented as frequencies and percentages, and continuous variables as means and standard deviations, or medians and interquartile ranges (25th to 75th percentile) for variables with skewed distributions. Increased cIMT was defined as a cIMT of 0.9 mm or higher [11]; hypertension as a systolic blood pressure (SBP) of 140 mm Hg or higher, or a diastolic blood pressure (DBP) of 90 mm Hg or higher or receiving antihypertensive treatment; diabetes mellitus as a FBG of 7.0 mmol/l or higher or receiving diabetes treatment; dyslipidemia as a serum TC>6.21 mmol/L, or LDL-C>4.14 mmol/L, or TG>1.70 mmol/L, or HDL-C<1.04 mmol/L; abdominal obesity as a waist circumference of 90 cm or higher for men and 80 cm or higher for women. The one-way analysis of variance (ANOVA) test was used to evaluate the differences in the parametric samples between the normal and increased cIMT groups, while the nonparametric Mann-Whitney test was used to determine significant differences when the data were not normally distributed. The associations of certain AAs with cIMT and other risk factors were assessed with the use of nonparametric Spearman correlation analyses. Stepwise multiple linear regression analysis was used to test the independent relationships of the measured variables with cIMT; meanwhile, collinearity testing was used to avoid including interdependent model variables. The significance levels for entering and removing an explanatory variable were set at 0.05 and 0.10, respectively. A stepwise logistic regression analysis was used to estimate the association between variables and subclinical atherosclerosis status, i.e., the presence or absence of increased cIMT. Odds ratios and 95% confidence intervals (CIs) for increased cIMT versus normal cIMT were calculated. Data were adjusted for age, sex, BMI, smoking status, presence or absence of diabetes, hypertension and dyslipidemias, family history of CAD, and therapies for diabetes, hypertension and dyslipidemias (hypercholesterolemia). All reported *P* values were two-tailed, with a *P* value of 0.05 indicating statistical significance. Analyses were performed with the use of SPSS software, version 16.0 (SPSS Inc.).

## Results

### Characteristics of the cross-sectional study population

The characteristics of the population are shown in **Table 1**. The most common characteristic was increased cIMT (72% of women, 85% of men). The prevalence of hypertension, diabetes, obesity, increased TG, TC and LDL-C, and decreased HDL-C were 59%, 18%, 12%, 27%, 9%, 7% and15%, respectively. Two percent of the women and 41% of the men were current or former smokers.

**Table 1:** Clinical characteristics of study population.

	Total (n = 472)	Men (n = 272)	Women (n = 200)
Age, years	70.1±6.6	70.2±6.0	69.9±7.4
BMI, kg/m^2^	24.4±3.3	24.67±3.03	24.01±3.61^a^
Waist circumference, cm	89.8±9.0	91.96±8.37	86.95±9.05^a^
Smoker, %	116 (24.6)	112 (41.1)	4 (2.0)^a^
Hypertension, %	280 (59.3)	161 (59.2)	119 (59.5)
Diabetes, %	83 (17.6)	52 (19.1)	31 (15.5)
BMI≥28, %	57 (12.1)	37 (13.6)	20 (10.0)
24≤BMI<28, %	194 (41.1)	120 (44.1)	74 (37.0)
Abdominal obesity, %	250 (53.0)	118 (43.4)	132 (66.0)^a^
Hypertriglyceridemia, %	127 (26.9)	56 (20.6)	71 (35.5)^a^
Hypercholesterolemia, %	41 (8.7)	16 (5.9)	25 (12.5)^a^
Hyper-LDL cholesterolemia, %	35 (7.4)	13 (4.8)	22 (11.0)^a^
Hypo-HDL cholesterolemia, %	69 (14.6)	47 (17.3)	22 (11.0)^a^
Diabetes therapy, %	75 (15.9)	47 (17.3)	28 (14)
Hypertension therapy, %	204 (43.2)	113 (41.5)	91 (45.5)
Cholesterol-lowering therapy, %	51 (10.8)	32 (11.8)	19 (9.5)
Family history of CAD, %	70 (14.8)	31 (11.4)	39 (19.5)^a^
Increased cIMT, %	375 (79.4)	231 (84.9)	144 (72.0)^a^

a *P*<0.05 compared with men.

BMI, body mass index; cIMT, carotid intima-media thickness.

### Univariate analyses

The concentrations of each AA showed skewed and leptokurtic distributions. As shown in **Table 2**, the increased cIMT group had significantly higher levels of Val, Ile, Leu, total BCAAs and Phe than the normal cIMT group, *P*<0.01. However, there were no significant differences for Tyr in the increased cIMT group compared to the normal cIMT group, though increment levels were observed. The normal and increased cIMT individuals differed significantly in age, BMI, WC, SBP and FBG concentration, *P*<0.05. Individuals with obesity and diabetes had significantly elevated BCAAs (*P*<0.01) and Phe levels (*P*<0.05).

**Table 2:** Univariate analyses of CAD risk factors with normal and increased cIMT.

	Normal cIMT (n = 97)	Increased cIMT (n = 375)	*P*
Age, years	68.2±6.4	70.6±6.5	0.002
BMI, kg/m^2^	23.6±3.5	24.6±3.2	0.013
WC, cm	83.7±9.3	87.6±8.7	<0.001
SBP, mmHg	130.3±18.2	135.9±17.6	0.007
DBP, mmHg	75.3±7.5	76.2±8.6	0.345
Val *, µmol/L	229.8 (208.9∼258.9)	248.8 (226.2∼275.3)	<0.001
Ile *, µmol/L	62.1 (56.9∼72.2)	69.1 (60.9∼78.6)	<0.001
Leu *, µmol/L	124.3 (110.8∼141.8)	133.3 (119.7∼147.5)	<0.001
BCAA *, µmol/L	413.4 (378.1∼472.7)	450.1 (407.5∼497.5)	<0.001
Tyr *, µmol/L	64.4 (59.4∼73.7)	65.7 (59.1∼72.7)	0.638
Phe *, µmol/L	74.4 (70.1∼80.4)	77.3 (71.3∼83.7)	0.006
AAA *, µmol/L	139.9 (129.3∼149.0)	143.0 (132.9∼156.1)	0.090
TC, mmol/L	5.0±0.9	5.0±1.0	0.735
TG *, mmol/L	1.3 (0.9∼1.7)	1.3 (0.9∼1.7)	0.553
HDL-C*, mmol/L	1.4 (1.1∼1.6)	1.3 (1.2∼1.6)	0.126
LDL-C *, mmol/L	2.9 (2.4∼3.3)	2.9 (2.3∼3.5)	0.786
Apo AI *, mg/dL	141.7 (130.8∼155.4)	138.9 (128.7∼150.5)	0.173
ApoB, mg/dL	96.6±20.0	98.4±21.4	0.445
hs-CRP *, mg/dL	0.1 (0.0∼0.2)	0.1 (0.0∼0.2)	0.777
Apo CII *, mg/dL	3.8 (2.5∼4.8)	3.9 (2.8∼5.4)	0.269
Apo CIII *, mg/dL	8.7 (7.4∼11.0)	9.1 (7.5∼11.2)	0.526
FBG *, mmol/L	5.3 (5.0∼5.7)	5.6 (5.2∼6.1)	0.001

*Median (Q1∼Q3).

CAD, coronary artery disease; cIMT, carotid intima-media thickness; BMI, body mass index; WC, waist circumference; SBP, systolic blood pressure; DBP, diastolic blood pressure; TC, total cholesterol; TG, triglycerides; HDL-C, high density lipoprotein cholesterol; LDL-C, low density lipoprotein cholesterol; hs-CRP, high-sensitivity C-reactive protein; FBG, fasting blood glucose. Apo AI, B, CII and CIII represent apolipoprotein AI, B, CII and CIII, respectively. Val, valine; Ile, isoleucine; Leu, leucine; Tyr, tyrosine; Phe, phenylalanine. BCAA represents the sum of the concentrations of Val, Ile and Leu; AAA represents the sum of the concentrations of Tyr and Phe.

### Correlation analyses

The relationships between certain amino acids and cIMT, and other parameters are shown in **Table 3**. In the nonparametric Spearman correlation analyses, Val, Ile, Leu, Phe and the total BCAA and AAA concentrations were significantly and positively correlated with cIMT (*P*<0.05). There was also a strong correlation between each amino acid and BMI (*P*<0.001) and waist circumference (*P*<0.001). Furthermore, all BCAA and AAA concentrations were significantly and positively correlated with TG and the apoB/apoAI ratio (*P*<0.001) and were inversely associated with HDL-C and apoAI (*P*<0.001). Ile and Leu were negatively correlated with TC (*P*<0.05). Positive correlations of BCAA and AAA concentrations with hsCRP, apoCII and apoCIII were also found. Serum BCAAs seemed to be negatively correlated with age, especially Leu (*P*<0.01). Positive correlations between all AAs and DBP (*P*<0.05) as well as Val and SBP (*P*<0.05), were observed. Most of BCAAs and AAAs were correlated with FBG and apoB. In addition, cIMT was significantly correlated with age (r = 0.228, *P*<0.001), BMI (r = 0.125, *P*<0.01), WC (r = 0.169, *P*<0.001), SBP (r = 0.218, *P*<0.001), FBG (r = 0.105, *P*<0.05), TG (r = 0.100, *P*<0.05), HDL-C (r = −0.125, *P*<0.01), apoAI (r = −0.116, *P*<0.05), and the apoB/apoAI ratio (r = 0.105, *P*<0.05). The BCAAs and AAAs were also strongly correlated with each other (*P*<0.001).

**Table 3:** Correlations (r) of amino acids with cIMT and other CAD risk factors.

	Val	Ile	Leu	Tyr	Phe	BCAA	AAA
cIMT	0.130 **	0.160 ***	0.137 **	0.046	0.153 **	0.144 **	0.110 *
Age	−0.083	−0.111 *	−0.139 **	−0.064	0.014	−0.108 *	−0.034
BMI	0.378 ***	0.369 ***	0.328 ***	0.387 ***	0.328 ***	0.373 ***	0.394 ***
WC	0.385 ***	0.411 ***	0.382 ***	0.347 ***	0.358 ***	0.401 ***	0.386 ***
SBP	0.110 *	0.075	0.054	0.032	0.041	0.089	0.043
DBP	0.190 **	0.197 **	0.177 ***	0.123 **	0.102 *	0.195 ***	0.127 **
FBG	0.150 **	0.151 **	0.177 ***	0.019	0.159 ***	0.161 ***	0.093 *
TC	−0.056	−0.145 **	−0.095 *	−0.008	−0.070	−0.088	−0.047
TG	0.269 ***	0.245 ***	0.248 ***	0.237 ***	0.223 ***	0.267 ***	0.261 ***
HDL-C	−0.382 ***	−0.444 ***	−0.406 ***	−0.332 ***	−0.333 ***	−0.413 ***	−0.378 ***
LDL-C	0.061	0.000	0.038	0.087	0.044	0.043	0.066
apoAI	−0.343 ***	−0.415 ***	−0.376 ***	−0.304 ***	−0.312 ***	−0.378 ***	−0.350 ***
apoB	0.140 **	0.066	0.100 *	0.142 **	0.104 *	0.117 *	0.136 **
apoB/apoAI	0.267 ***	0.239 ***	0.251 ***	0.249 ***	0.225 ***	0.265 ***	0.267 ***
apoCII	0.146 **	0.125 **	0.151 **	0.091	0.093 *	0.150 **	0.106 *
apoCIII	0.118 *	0.053	0.097 *	0.018	0.093 *	0.103 *	0.064
hsCRP	0.206 ***	0.134 **	0.160 ***	0.178 ***	0.319 ***	0.180 ***	0.269 ***

*, *P*<0.05; **, *P*<0.01; ***, *P*<0.001.

CAD, coronary artery disease; cIMT, carotid intima-media thickness; BMI, body mass index; WC, waist circumference; SBP, systolic blood pressure; DBP, diastolic blood pressure; TC, total cholesterol; TG, triglycerides; HDL-C, high density lipoprotein cholesterol; LDL-C, low density lipoprotein cholesterol; hs-CRP, high-sensitivity C-reactive protein; FBG, fasting blood glucose. Apo AI, B, CII and CIII represent apolipoprotein AI, B, CII and CIII, respectively. Val, valine; Ile, isoleucine; Leu, leucine; Tyr, tyrosine; Phe, phenylalanine. BCAA represents the sum of the concentrations of Val, Ile and Leu; AAA represents the sum of the concentrations of Tyr and Phe.

### Multiple linear regression model

For multivariate reevaluation of the univariate correlations, all variables given in **Table 3** were entered into a stepwise multiple linear regression analysis as independent variables to identify significant contributors to the distribution of cIMT. The stepwise multiple linear regression analysis revealed that age (β = 0.175, *P*<0.001), log BCAA (β = 0.147, *P*<0.001) and SBP (β = 0.141, *P* = 0.012) were independent factors that correlate with cIMT (adjusted R^2^ = 0.041, *P* = 0.002) as shown in **Table 4**.

**Table 4:** Multiple linear regression analysis with cIMT as the dependent variable.

Variables	Regression coefficient	SE	β	*P*
Age	0.011	0.003	0.175	<0.001
Log BCAA	0.937	0.295	0.147	<0.001
SBP	0.003	0.001	0.141	0.012

β indicates the standardized partial regression coefficient.

All variables given in Table 4 were entered into a stepwise multiple linear regression analysis as independent variables. Collinearity testing was used to avoid including interdependent model variables. *P* values for entry and removal, 0.05 and 0.10, respectively.

### Logistic regression model

The results of the logistic regression analysis of the differences between the increased and normal cIMT groups are shown in **Table 5**. Although the univariate models demonstrated significant differences in several variables between increased cIMT and normal cIMT groups (**Table 2**), many of them did not appear in the model adjusted for age, sex, BMI, smoking, BCAA quartile, AAA quartile, diabetes mellitus, hypertension, dyslipidemias, family history of CAD, and diabetes, hypertension and cholesterol-lowering therapies. In the logistic regression model, BCAA was the most and sole powerful laboratory factor correlated with increased cIMT (the odds ratio of the fourth quartile compared to the first quartile was 2.679; P = 0.009).

**Table 5:** Logistic regression model for the cross-sectional population.

Independent variables of increased cIMT^a^	B^b^	S.E.	P	Exp(B)	95.0% C.I.for EXP(B)
Age	0.059	0.021	0.004	1.060	1.019∼1.104
Men	−0.660	0.270	0.015	0.517	0.305∼0.878
BCAA First quartile			0.005		
Second quartile	1.057	0.355	0.003	2.879	1.436∼5.771
Third quartile	0.887	0.346	0.010	2.427	1.233∼4.777
Fourth quartile	0.985	0.376	0.009	2.679	1.281∼5.603
Hypertension	0.868	0.261	0.001	2.383	1.429∼3.972

a Set of independent variables: age, sex, BMI, smoking, diabetes mellitus, hypertension, hypertriglyceridemia, hypercholesterolemia, hyper-LDL cholesterolemia, hypo-HDL cholesterolemia, diabetes therapy, hypertension therapy, cholesterol-lowering therapy, BCAA quartile and AAA quartile and family history of CAD.

b B, coefficient; SE, standard error of B; exp(B), estimated odds ratio.

## Discussion

Recent studies have consistently shown that BCAAs and AAAs are strongly correlated with insulin resistance [3]–[5], diabetes development [6], [7] and intervention outcomes [12]–[14] and are uniquely responsive to therapeutic interventions. Some other investigations have demonstrated that circulating levels of BCAAs and AAAs are not only associated with obesity and impaired glucose tolerance but also with dyslipidemia [15]. One of our previous studies also found that BCAAs and AAAs were related to atherogenic dyslipidemia [8], which is characterized by an increase in serum TG, a decrease in HDL-C and the prevalence of small-dense LDL [16]. The state of dyslipidemia, which often precedes diabetes, is associated with an increased risk of developing atherosclerosis and CAD [9]; thus, it is of interest to explore the associations of BCAAs and AAAs with the progress of atherosclerosis.

There have been a few studies on the association between certain amino acids and CAD. Shah and colleagues [17] used targeted LC-MS to profile 69 metabolites in individuals with and without CAD that had been referred to an academic cardiac catheterization laboratory. BCAAs were positively and independently associated with the presence of CAD (OR, 1.36; *P* = 0.02). However, they subsequently found that BCAAs were negatively associated with all-cause death (232 cases, HR, 0.86; *P* = 0.03) and all-cause death plus MI (294 cases, HR, 0.88; *P* = 0.05) in a cohort study with a median follow-up of 3.1 years [18]. Subjects in the two studies were all patients undergoing cardiac catheterization and the CAD prevalence was high (∼60% with 1–3 diseased vessels). The seemingly discrepant results in the two studies may be related to the fact that older people are more susceptible to all-cause death and BCAAs are likely to be negatively associated with age (discussed below). BCAAs would still be positively, or at least not negatively, associated with atherosclerosis related diseases as the negative correlation became weaker when MI was included (HR 0.88 vs. 0.86). Recently, Magnusson and colleagues [19] reported on a Swedish population based cohort study and found that an amino acid score including branched-chain and aromatic amino acids (Ile, Tyr and Phe) predicted cardiovascular diseases during 12 years of follow-up and was independently correlated with cIMT. This study clearly indicates that certain amino acids are early markers of atherosclerotic CAD development.

In the present study, we explored the association of serum amino acids with atherosclerosis in randomly selected middle-aged and older Chinese subjects. It is well-established that cIMT reflects subclinical atherosclerosis and is an important risk factor for CAD [10], [20]–[22]. The cIMT values of 472 subjects were assessed by B-mode ultrasonography and the subjects were categorized into increased and normal cIMT groups using a cut-point of 0.9 mm [11]. Serum BCAAs and AAAs in the subjects were measured with our previously established LC/MS/MS method. Serum levels of BCAAs and AAAs except for Tyr in the increased cIMT group were significantly higher than those in the normal group. The correlation analyses also showed strong associations of BCAAs and AAAs with cIMT. The relationship between BCAAs and cIMT was still present in the multivariate linear regression analysis, which controlled for traditional CAD risk factors. Total BCAA concentrations, along with age and SBP were independent factors that contributed to explaining the variance in cIMT and individuals with increased cIMT tended to be older and have higher BCAA levels. Among all biochemical markers assessed, BCAAs were the one that were correlated most with increased cIMT. Taken together, the current study demonstrates that a higher level of BCAAs is positively and independently associated with increased cIMT. Our findings are consistent with that of Magnusson et al [19] although our study sample is apparently different in ethnicity, BMI, and probably other culture-related aspects, such as lifestyle and diet.

In addition, we also analyzed the correlations of BCAAs and AAAs with other clinical and laboratory characteristics and the results were consistent with prior studies. Felig and colleagues [23] originally reported that BCAAs and AAAs correlated with obesity and serum insulin. More recent investigations, as well as our previous research, highlighted associations of certain amino acids with obesity, elevated serum glucose, dyslipidemia and high blood pressure [3]–[5], [8], [15]. The present study extends the prior work by demonstrating robust and reproducible associations of circulating levels of BCAAs and AAAs with these previously studied risk factors. Furthermore, the present study also demonstrated significant correlations of BCAAs and AAAs with inflammation related markers, such as hsCRP, and some other apolipoproteins, such as apoCII and apoCIII. It should also be noted that this study showed negative correlations between BCAAs and age. Further studies should be undertaken to determine and evaluate the mechanisms behind these relationships.

Several limitations of this study deserve mentioning. First, we did not analyze the effect of individual BCAAs and AAAs, but used the sum of the BCAAs and AAAs because of their strong intercorrelations which may have influenced the accuracy of explanation of the model. Second, the study population was comprised of predominantly middle-aged to elderly individuals. The generalizability of our findings to younger individuals is not yet known. Third, we did not record sedentary lifestyle, stress/depression and other possible risk factors of atherosclerosis in the subjects. We did not either record the details of the medications. These factors may also affect the observed correlations. Finally, this is a cross-sectional study of small sample size and the findings need to be confirmed in future studies.

In conclusion, using a rapid and precise LC/MS/MS method to measure serum BCAAs and AAAs, we demonstrated obvious associations of BCAAs and AAAs with cIMT, BMI, lipid profiles, blood pressure and inflammation risk factors. These associations would open a new field for the mechanistic understanding of atherosclerosis and the risk assessment of CAD. Further investigation are warranted to test whether plasma amino acid measurements can help predict subclinical atherosclerosis or CAD and to elucidate the biological mechanisms of the identified associations between plasma amino acids levels and various CAD risk factors.
